# Multi-colony tracking of a marine central place forager reveals a site specific yet broadly consistent foraging strategy

**DOI:** 10.1007/s00227-026-04909-3

**Published:** 2026-07-15

**Authors:** Jonathan M. Handley, Joshua C. Wilson, Norman Ratcliffe, Alastair M. M. Baylis, Sarah Crofts, Pierre A. Pistorius

**Affiliations:** 1 Marine Apex Predator Research Unit, Department of Zoology and Institute for Coastal and Marine Research, Nelson Mandela University, South Campus, Port Elizabeth, 6031 South Africa; 2 School of Ocean and Earth Science, University of Southampton, National Oceanography Centre, European Way, Southampton, SO14 3ZH UK; 3 British Antarctic Survey, High Cross, Madingley Road, Cambridge, CB3 0ET UK; 4 South Atlantic Environmental Research Institute, FIQQ1ZZ Stanley, Falkland Islands; 5 Wild Falklands Ltd., FIQQ 1ZZ Sea Lion Island, Falkland Islands; 6 Falklands Conservation, FIQQ 1ZZ Stanley, Falkland Islands

**Keywords:** GPS, TDR, Gentoo penguin, Hurdle model, Foraging ecology, Falkland Islands

## Abstract

Colonial central place foraging seabirds with a wide species range inhabit highly heterogenous habitats, which may result in colony-specific foraging behaviours. Simultaneously tracking seabirds from multiple colonies is key to elucidating factors influencing foraging behaviour and determining how species might respond to environmental perturbations. Here, we investigate the foraging behaviour of gentoo penguins (*Pygoscelis papua*) breeding at the Falkland Islands in relation to habitat availability and prey types. We examine the horizontal and vertical foraging distribution of 78 birds during two breeding seasons from three colonies, selected for their geographic separation and contrasting habitats. Through comparison of general foraging characteristics, we found that at each colony, penguins exhibited either pelagic foraging behaviour in the water column, or benthic foraging behaviour near to the sea floor. This was dictated by the surrounding bathymetry, with pelagic behaviour being associated with steeper surrounding slopes, and was in accordance with the known diet of birds. Then, using a hurdle model to predict dive intensity around each colony, we show that habitat suitability for all colonies declined once distance to the colony exceeded ~ 30 km, while other variables had differing influences. At different colonies, the use of either pelagic or benthic foraging behaviours suggests that gentoo penguins have the capacity to respond to variable environmental conditions. However, given that foraging trips only reached mean maximum distances of 23.1 km ± 12.2 km from the colony during the breeding season, marine spatial planning efforts need to protect inshore waters around critical gentoo penguin colonies to effectively conserve the species.

**Supplementary Information:**

The online version contains supplementary material available at 10.1007/s00227-026-04909-3.

## Introduction

Central place foraging seabirds with broad geographic ranges may exhibit variable foraging behaviours across heterogenous local environments (Corman et al. 2016; Quillfeldt et al. 2019). The factors dictating variability in foraging behaviours and the overall foraging strategy of a species can be complex, spanning a range of abiotic factors such as coastal morphology (Lescroël and Bost 2005) and oceanic conditions (Gilmour et al. 2018), and biotic factors such as prey availability (Gulka et al. 2020), density dependent competition (Trevail et al. 2023), and predator avoidance (Masello et al. 2017). Understanding the factors that influence seabird foraging strategies provides critical insights into how species may respond to environmental changes, including those driven by climate change, and can guide the development of effective conservation actions (Hays et al. 2016; Orgeret et al. 2022).

During the breeding season, colonial breeding seabirds need to return to their nests to provision chicks or relieve partners regularly, which constrains their foraging trip duration (Oppel et al. 2018). Hence, the breeding season provides a useful temporal window to explore variation in foraging behaviours, given that seabird breeding colonies in different locations typically have discrete colony specific foraging areas that are often associated with contrasting marine habitats (Cleasby et al. 2024). For example, Fiordland penguins (*Eudyptes pachyrhynchus*) were tracked from two colonies 8 km apart in the same fjord, and in a single year, those from one colony mostly foraged within the fjord, while those from the other colony made longer foraging trips to open water (Otis et al. 2025). Similarly, a multi-year, multi-colony tracking study of Manx shearwaters (*Puffinus puffinus*) found that birds often forage within the local vicinity of their colonies, but also sometimes travel large distances to a single shared area (Dean et al. 2015). This variation in foraging behaviour of seabirds with respect to colony location has also been shown in different use of the water column by species, with birds from different colonies foraging at different depths (Muller et al. 2020; Divoky et al. 2021; Legard et al. 2025).

The gentoo penguin (*Pygoscelis papua*) is a central place foraging seabird that inhabits a vast geographic range (Ratcliffe and Trathan 2012), and represents a useful model species to assess variability in foraging behaviours. Biologging studies have revealed that gentoo penguins consistently forage within a small radius around their colonies (~ 30 km), leading to a wide array of foraging behaviours depending on local habitat and prey availability across the South Atlantic Ocean (Masello et al. 2017; Ratcliffe et al. 2018; Baylis et al. 2019; Harris et al. 2020, 2023), subantarctic Indian Ocean (Lescroël and Bost 2005; Carpenter-Kling et al. 2017; Camprasse et al. 2017), and the Antarctic Peninsula (Miller et al. 2009; Hinke et al. 2017; Lee et al. 2021; Veatch et al. 2025). The Falkland Islands in the South Atlantic form a site of particular importance, hosting approximately 30% of the global population (Herman et al. 2020). Genetic evidence also suggests that gentoo penguins in the Falkland Islands may form a distinct species (Tyler et al. 2020; Noll et al. 2026), highlighting the importance of this region.

The diet of Falkland Islands gentoo penguins has been well-studied and shows that diet composition varies across colonies, which likely reflects the local availability of prey around breeding colonies (Pütz et al. 2001; Clausen et al. 2005). In contrast, biologging studies are limited (Boersma et al. 2002; Masello et al. 2010, 2017; Baylis et al. 2020), with at-sea foraging behaviours during the breeding season being poorly understood. At New Island in the Falkland Islands, birds from two nearby colonies forage with little to no overlap in their at-sea areas, despite both using inshore waters (Boersma et al. 2002; Masello et al. 2010, 2017). Recent studies also support the notion of colony differences in foraging behaviour (Handley et al. 2018; Baylis et al. 2019), and inference about foraging behaviours from these studies would be improved with knowledge of species distribution at sea from telemetry data (Carneiro et al. 2024). There are 75 gentoo penguin colonies across the Falkland Islands (Handley et al. 2023), and there has not been a comprehensive assessment of at-sea distribution from across the archipelago. Understanding how foraging behaviours may vary geographically will enhance our knowledge of the foraging ecology of this species in a region hosting a significant proportion of the global population. This knowledge can ultimately support conservation and management actions for this species with knowledge on at-sea distribution being a key consideration for marine spatial planning (Baylis et al. 2021; Handley et al. 2023).

Here, using telemetry data, we explore the foraging behaviour of gentoo penguins at the Falkland Islands during the breeding period (November to January) to enhance understanding of the foraging ecology of these birds beyond diet studies alone. Using GPS loggers and time-depth recorders (TDRs), we tracked birds over two breeding seasons from three colonies which were selected for their geographic isolation from each other and differences in the marine habitats within their typical foraging ranges from elsewhere. This is the first study to track individuals from multiple colonies across different regions of the archipelago, providing a comprehensive assessment of at-sea behaviour. We aimed to enhance understanding of the foraging strategy of gentoo penguins across the Falkland Islands, and any causes of variation in foraging behaviours of this central place foraging species. These analyses are crucial given the global importance of the Falkland Islands for gentoo penguins and the possibility of the Falkland Islands gentoo penguin being a distinct species. Such data are essential to inform conservation measures - such as establishing Marine Protected Areas (MPAs) – and can help mitigate other threats and support population resilience.

## Methods

### Study sites

Birds were sampled at three colonies on the Falkland Islands during the austral summers of 2012/13 and 2013/14, hereafter referred to as 2012 and 2013. These colonies were Steeple Jason Neck (51.0375° S, 61.2097° W), Cow Bay (51.4288° S, 57.8703° W), and Bull Roads (52.3096° S, 59.3896° W). The number of breeding pairs at the time of sampling were 3710 at Steeple Jason Neck, 1821 at Cow Bay, and 1236 at Bull Roads (Stanworth 2013). Each colony has unique surrounding bathymetry and exposure to the open ocean. Steeple Jason Island is situated in the open ocean and has a steeply sloping seabed that rapidly drops down to 100 m. Cow Bay, which faces the open ocean, and Bull Roads, which is in a sheltered shallow bay, both have seabeds that gradually slope away. All colonies are single-species colonies though other species breed near to Cow Bay and Steeple Jason Neck (Kuepfer and Stanworth 2024). Steeple Jason has large breeding populations of black-browed albatross (*Thalassarche melanophris*), southern rockhopper penguins (*Eudyptes chrysocome*), and imperial shags (*Leucocarbo atriceps*). At Cow Bay, Magellanic penguins (*Spheniscus magellanicus*) breed in burrows distributed along the coastline, and near to Cow Bay (~ 5 km), Volunteer Point hosts the largest breeding colony of king penguins (*Aptenodytes patagonicus*) at the Falkland Islands. There are no notable colonies of other seabirds in proximity to Bull Roads. In 2013, waters around the Falkland Islands were generally warmer and exhibited greater chlorophyll-*a* concentrations than those of 2012, though the waters around Cow Bay did not display notable differences in chlorophyll-*a* concentration (Supplementary Fig. 1; Supplementary Table 1).

### Logger deployment

To reduce possible biases in foraging parameters owing to varied clutch or brood size (Williams and Rothery 1990), loggers were deployed on birds incubating two eggs or guarding two chicks. For incubating birds, devices were only deployed once two eggs had been laid and both partners had completed a foraging trip. Loggers were first deployed on birds incubating eggs at Steeple Jason between October 29th and November 7th 2012, during an opportunity to tag birds alongside the annual seabird census efforts across the Falkland Islands. Birds guarding chicks were then tagged over two seasons at Cow Bay (November 27th to December 23rd 2012, December 15th to December 28th 2013) and Bull Roads (November 21st to December 9th 2012, December 2nd to December 9th 2013).

Each logger was secured separately to the feathers on the midline of the back of the bird using overlapping layers of waterproof adhesive TESA^®^ tape (Beiersdorf, AG, GmbH, Hamburg, Germany) that were sealed with cyanoacrylate glue (Loctite 401^®^). Any tape above the GPS antennae was removed, otherwise satellite signals would not be received when the tape becomes wet.

Handling time was kept to a minimum, typically below 15 min and always below 20 min. A continual watch was maintained between 11:00 and 23:00 for returning birds so they could be recaptured away from the colony to minimise disturbance. Average logger deployment time was 22.21 h (± 14.03 h).

Three types of GPS device were used during the study: The Earth & OCEAN Technology 2-AA model (140 × 39 × 26.5 mm, 147 g), the SIRTRACK^®^ Fastloc 2 model (69 × 28 × 21 mm, 39 g) and the Catnip Technology CatTraQ GPS logger (44 × 27 × 13 mm, 22 g). Pressure (bar) and temperature (°C) data were recorded with a CEFAS G5 TDR (31 × 3 mm, 2.7 g). The maximum weight of device combinations, including cameras when used (Handley and Pistorius 2016), never exceeded 172.7 g, approximately 2.7% of the body mass of a bird. Tags of this size have been placed on gentoo penguins elsewhere and have been shown to have negligible effects on the foraging behaviour of the penguins (Ratcliffe et al. 2018).

GPS units were programmed to maximise positional fix rates while still capturing a complete foraging trip. The 2-AA and CatTraQ units were programmed to record locations every minute. Additionally, the 2-AA unit was set with a 10-minute fall back time and the pressure control disabled. This causes the unit to search for satellites at one-second intervals when no fix is obtained and is beneficial for diving animals with short surface intervals. If locations are recorded continuously for 10 min (e.g. when the animal is on land or continually at the surface) the unit falls back to one-minute intervals, avoiding excessive battery use. The Fastloc 2 unit employs a snapshot technique different to traditional GPS fixing, and requires post processing of data with SIRTRACK^®^ software, which reduces fix acquisition times and maximises the likelihood of obtaining positions during short surface intervals between dives. The units were set to record fixes at two-minute intervals due to battery limitations. No differences in trip characteristics were detected at the *p* < 0.05 level among the different types of GPS device used here. TDR loggers were programmed to sample pressure and temperature every second.

### Foraging characteristics

Data were analysed using R version 4.5.1 (R Core Team 2025). Summary statistics of dive behaviour were determined from TDR data using the *diveMove* R package v1.6.4 (Luque 2024). Dives were limited to a threshold of at least 3 m depth to disregard shallow travelling dives following other studies (van Eeden et al. 2016; Lescroël et al. 2021; Oosthuizen et al. 2025). Summary statistics included the dive duration (time spent underwater on each dive), maximum dive depth (the maximum water depth reached during a single dive), and bottom time (duration of the bottom phase of a dive when a bird maintains a relatively consistent depth). The package allowed for an accurate time budget summary of bird departure to sea and return to land. This summary was amended, if needed, through visually inspecting the temperature trace for a rapid change in ambient temperature. Owing to sensitivities of the transducer within TDRs, pressure readings often need to be zero offset corrected, i.e. if a device consistently records below or above sea level (expected to be 0 m depth), the data requires correction. This was achieved manually for all deployments through visual inspection of the pressure trace during the calibration step. Dives were classified as benthic if the maximum depth of a series of dives was uniform and the difference was within ± 10% of the maximum depth reached during the preceding dive; suggesting, because of a lack of deeper dives, that the sea floor was the limit (Carpenter-Kling et al. 2017). Otherwise, dives were classified as pelagic.

CatTraQ GPS loggers tended to duplicate locations at sea, so these duplicates were removed. The *trip* package v1.10.0 (Sumner et al. 2015) was used to remove erroneous locations when the average transit speed between them was over 8 kmh^− 1^ (Adams and Wilson 1987). Furthermore, wave wash and diving can interrupt GPS fix acquisition and result in gaps in tracks that are much longer than the user defined schedule. Therefore, filtered data were processed with the *crawl* R package v2.3.0 (Johnson et al. 2008), using a continuous-time correlated random walk model to generate the most likely path used by a bird through generation of 100 possible tracks. Locations along each generated track were interpolated at one-minute intervals and averaged to produce a best-fit path. The total path length (distance travelled along the entire trip), maximum distance from the colony (furthest distance between a single trip location and the colony of origin), trip duration (the time between leaving the colony and returning to the colony), and average travelling speed (the mean velocity between track locations) were calculated using the *move* R package v4.2.6 (Kranstauber et al. 2024). Furthermore, from the 100 possible tracks, estimates of uncertainty in the path followed between GPS positions could be accounted for.

Using the dive locations along the best-fit tracks, we followed a similar approach to Lascelles et al. (2016) to determine the representativeness of samples compared with that of the sample population. Specifically, kernel density distributions representing the 50% utilisation distribution (UD) were generated for each colony during each stage in a given year using the *adehabitatHR* R package v0.4.22 (Calenge 2024) to identify the area of core use for the sample population. A bootstrap approach (*n* = 500) was then used to compare the overlap between the area used by the sample population versus randomly selected tracks, where the number of randomly selected tracks was increased iteratively from 1 to n_tracks_ − 1 for a given sample population. The smoothing parameter, h, was determined using Silverman’s ad-hoc method (Silverman 1998), as the least-squares cross validation method failed to converge. To quantify spatial overlap between birds from different colonies or breeding seasons, we calculated the Bhattacharyya’s Affinity of UDs. This metric ranges from 0 (entirely separate distributions) to 1 (identical distribution), based on the shape and alignment of distributions.

To further ensure that samples were representative of the trip characteristics of the colony, we took the data from the two colonies with the greatest sample sizes (Bull Roads and Cow Bay) and created 1000 bootstrapped samples of these data with only eight individuals (equivalent to the number of individuals from Steeple Jason). We then calculated the mean for each trip characteristic for each bootstrapped sample and calculated what percentage of samples fell within one true standard error and standard deviation of the full sample.

Linear mixed effects models were fit with the *nlme* R package v3.1-168 (Pinheiro et al. 2025) to compare dive and trip characteristics derived from TDR and GPS devices, following Zuur et al. (2009). All models featured normal errors and an identity link, using colony and year as interactive fixed effects to account for possible interannual variability in characteristics. To account for heterogeneity in the data across colonies (per stratum), models were weighted with the *varIdent* variance structure. Furthermore, temporal autocorrelation was observed for all dive variables, so these models were fitted with a first-order autocorrelation structure. Trip ID was used as a random effect for diving variables (maximum depth, bottom time, and dive duration) and bird ID was used as a random effect for trip characteristics (trip duration, maximum distance from the colony, path length, average speed). Models were compared with null models to test whether colony had a substantial effect on characteristics, using the Akaike information criterion (AIC), Bayesian information criterion (BIC), and maximum log-likelihood (Supplementary Table 2). Tukey HSD post-hoc pairwise comparisons between different colonies and years were performed to determine where differences were statistically significant using the *emmeans* R package v2.02 (Lenth and Piaskowski 2026).

### Habitat use

In addition to comparing foraging characteristics among colonies, we wanted to investigate which spatial factors might influence the diving intensity of birds with respect to their surrounding habitat. Using dive locations rather than area restricted search (ARS) locations more effectively discriminates resting and foraging behaviour in diving animals, which are difficult to distinguish from step lengths and turning angles alone (Weimerskirch et al. 2007).

Using the 100 *crawl* generated tracks and their associated dive records for each bird, we gridded the estimate of dive effort per cell (0.005° × 0.005°). Each grid was summed across all birds to produce a relative surface of dive effort for the sampled population. Gridding the uncertainty in locations accounts for their precision and also provides some degree of smoothing compared to simple gridding of the most likely primary positions (Ratcliffe et al. 2014). Although we used a degree-based grid with variable cell area, the narrow latitudinal range of this study meant that the actual cell area only varied between 427 m and 446 m.

An important prerequisite in determining habitat use by an animal is to determine the area available to that animal (Matthiopoulos et al. 2023). This available area is an indication of where animals could have travelled should they not have any preference in foraging habitat. A broad array of approaches have been used to define this area (Hazen et al. 2021). We chose a track simulation approach (Raymond et al. 2015), implemented with the *availability* R package v0.12 (Raymond et al. 2016). Through this method, habitat availability was estimated by simulating 100 tracks per observed track. Simulated tracks were bounded by a land mask and constrained in their trip durations, turning angles, travel speeds, and departure locations to resemble the movement characteristics of observed tracks. The available habitat was then considered to be the area encompassed by the maximum and minimum latitude and longitude reached across all simulated tracks combined. Absences were defined as cells within this area not used by the birds.

Satellite derived dynamic variables such as sea surface temperature, sea surface height, chlorophyll-*a* concentration, eddy kinetic energy, and sea level anomaly are often used as proxies for prey availability, a likely driver of animal habitat selection (Raymond et al. 2015; Hindell et al. 2020; Reisinger et al. 2022). A key requirement for habitat selection models is that the predictor variables are at an appropriate extent and resolution to capture the processes that influence selection (Araújo et al. 2019). Because of the short foraging range of gentoo penguins and the coarse spatial resolution of satellite imagery, the variability of dynamic predictors within this range was not considered substantial enough to be biologically meaningful (Supplementary Fig. 1; Supplementary Table 1). Further, incorporating dynamic predictors degraded model performance scores (Supplementary Table 3). Therefore, we only included the static variables of depth, slope, distance to the coast, and distance to the colony (a land mask cost surface was used when determining this distance, accounting for barriers such as headlands and islands). Depth was obtained from the General Bathymetric Charts of the Oceans at a 15 arc second resolution (GEBCO, 10.5285/37c52e96-24ea-67ce-e063-7086abc05f29), and slope was derived from depth using the *terrain* function in the *terra* R package v1.9-1 (Hijmans et al. 2026). Depth and slope were then resampled through bilinear interpolation to the 0.005° resolution of the dive effort rasters. Distance rasters were also created at this resolution. Data from the TDR devices and evidence from video footage (Handley et al. 2018) showed birds to be performing benthic dives at two sites where gentoo penguins feed primarily on benthic prey (Handley et al. 2017), suggesting that bathymetric variables are likely to be pertinent drivers.

To model the relationships between environmental variables and diving intensity, we fit a two-part hurdle model (Zuur et al. 2009). In this approach, an initial model is used to predict the probability of presence of gentoo penguin diving. All cells where diving behaviour was recorded are given a value of 1 and all other available cells are given a value of 0, modelling the probability of presence as a binomial response to environmental variables. A second model then estimates the relationship between diving intensity and environmental variables within cells where penguins were present. Predicted probability of presence (Supplementary Fig. 2) and diving intensity (Supplementary Fig. 3) are then multiplied to produce final estimates of the distribution of diving intensity around each colony. We fit separate hurdle models for each colony to investigate how species-habitat associations differ among colonies.

Boosted regression trees (BRTs) were chosen to fit the hurdle model, since they are robust machine learning algorithms, able to cope with outliers, non-linear relationships, and collinearity among variables (Elith et al. 2008; Torres et al. 2015). A further advantage of BRTs is that they can handle sharp discontinuities, which often occur when modelling the distributions of species that occupy only a small proportion of the sampled environmental space (Elith et al. 2008), as was evident in our data. BRTs were implemented with the *tidymodels* R framework v1.3.0 (Kuhn and Wickham 2020) and the *tidysdm* R package v1.0.0 (Leonardi et al. 2024), using *lightgbm* as an engine (Ke et al. 2017). BRT hyperparameters were optimised using 10-fold random cross-validation, varying the learning rate between 0.005 and 0.01, the tree depth between 1, 2, and 3, and the number of trees between 500, 1000, 1500, and 2000. Variable importance was determined through the relative contribution of a variable during model building. Partial dependence plots (PDPs) were then used to depict the effect of a variable on the response after accounting for the average effects of all other variables in the model. As a probabilistic model, the presence-absence model was evaluated with the Continuous Boyce Index (CBI), Area Under the Receiver Operating Curve (AUC) and True Skill Statistic (TSS). As a regression model, the diving intensity model was evaluated with Mean Absolute Error (MAE), Root Mean Squared Error (RMSE) and the Coefficient of Determination (R^2^).

## Results

### Data outcome and quality

In total, 103 birds were equipped with both GPS and TDR units during the study. 85.4% of loggers were successfully recovered (*n* = 88), since the batteries from two deployments failed, four birds were not recaptured, and nine devices failed owing to water penetration (Supplementary Table 4). Typically, birds were recaptured after a single trip but in some cases only after a second trip (*n* = 13). Birds were sampled during the incubation period of 2012 for all three colonies, and during the guard period of 2012 and 2013 for Cow Bay and Bull Roads. The check for sample representativeness indicated that too few tracks were obtained for the incubation period of 2012 at Cow Bay (*n* = 5) and Bull roads (*n* = 5), since accumulation curves did not reach the 95% probability of overlapping threshold, or only reached this threshold upon including the final individual (Supplementary Fig. 4). Therefore, after first testing for inter-seasonal differences at Bull Roads and Cow Bay, the across colony comparison was made among 91 trips made by the remaining 78 successful deployments from the 2012 incubation period at Steeple Jason (n_birds_ = 8), and the combined 2012 and 2013 guard periods at Cow Bay (n_birds,2012_ = 17, n_birds,2013_ = 19) and Bull Roads (n_birds,2012_ = 14, n_birds,2013_ = 20). When resampling the number of birds from Cow Bay and Bull Roads to eight individuals, 69.2% of bootstrap sample trip characteristic means fell within one standard error and 99.9% fell within one standard deviation of the full sample mean (Supplementary Fig. 5).

### At-sea distribution

**Fig. 1:**
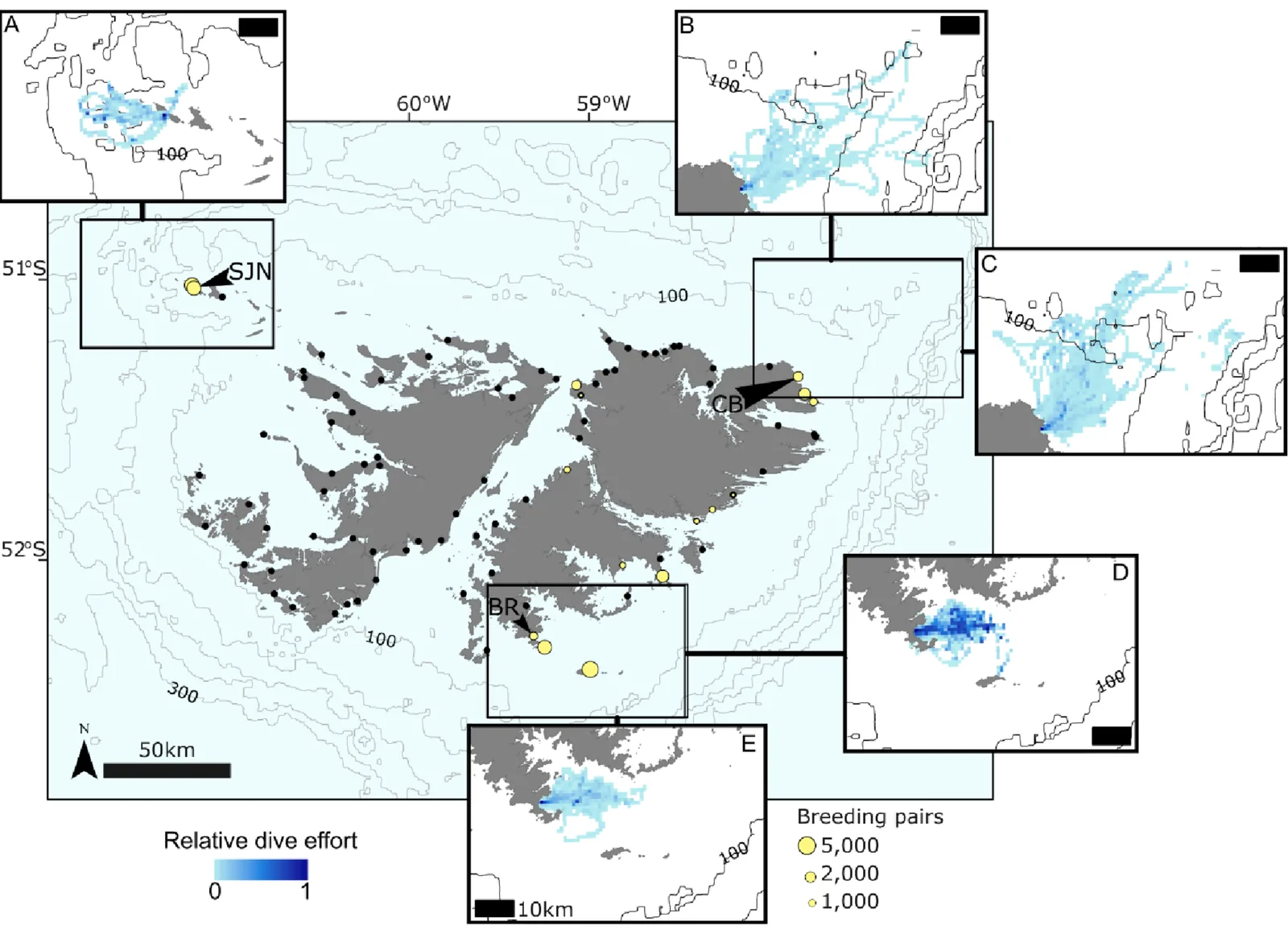
Distribution of gentoo penguins at the Falkland Islands during the 2012 and 2013 breeding seasons. Insets include the (**A**) 2012 incubation period of Steeple Jason Neck (SJN), the (**B**) 2012 and (**C**) 2013 guard period of Cow Bay (CB), and the (**D**) 2012 and (**E**) 2013 guard period of Bull Roads (BR). Cell colours in the insets indicate relative dive effort per cell from all tracks combined. The black bar represents a 10 km scale bar. Known extant gentoo penguin colonies are indicated by black dots, while yellow dots indicate the size of annually monitored colonies during the 2012 breeding pair census (Stanworth 2013). The 100 m and 300 m isobaths are labelled, and other isobaths are at 50 m intervals between these values. Maps were created in ArcGIS Pro v3.5 (ESRI 2025). The Falkland Islands shapefile was obtained from the South Atlantic Environmental Research Institute (https://www.south-atlantic-research.org/webgis-page/)

In all cases, birds foraged inshore, over the continental shelf (Fig. 1). Penguins from Cow Bay spread out from the colony in a northeasterly direction over the shelf (Fig. 1), in waters with an average seafloor depth of 83.3 m (± 29.7 m). These birds used the largest areas at sea, comprising 893 km^2^ and 1054 km^2^ in the 2012 and 2013 guard period, respectively. Similar areas were used in both years (Bhattacharyya’s Affinity = 0.897). Birds from Bull Roads used a smaller area, encompassing only 299 km^2^ in 2012 and 279 km^2^ in 2013. This area was located within the adjacent shallow bay during the guard period (Fig. 1), over mean seafloor depths of 33.2 m (± 19.7 m). Again, there was considerable overlap in the used areas across both seasons (Bhattacharyya’s Affinity = 0.974). During the 2012 incubation period, penguins from Steeple Jason Neck only used an area of 284 km^2^ to the west of the colony (Fig. 1), in waters with mean seafloor depths of 76.3 m (± 26.8 m). The seafloor depths used by birds from Bull Roads were shallower than those from Cow Bay (t = 11.84, *p* < 0.0001) or Steeple Jason (t = 7.51, *p* < 0.001). Conversely, birds from Steeple Jason navigated waters with steeper slopes of 0.79° (± 0.56°) than the waters used by penguins from Cow Bay (0.26° ± 0.29°; t = 10.58, *p* < 0.001) and Bull Roads (0.38° ± 0.16°; t = 9.16, *p* < 0.001). No overlap was seen in the areas used by the different colonies (Supplementary Fig. 6).

### Dive and trip characteristics

**Fig. 2:**
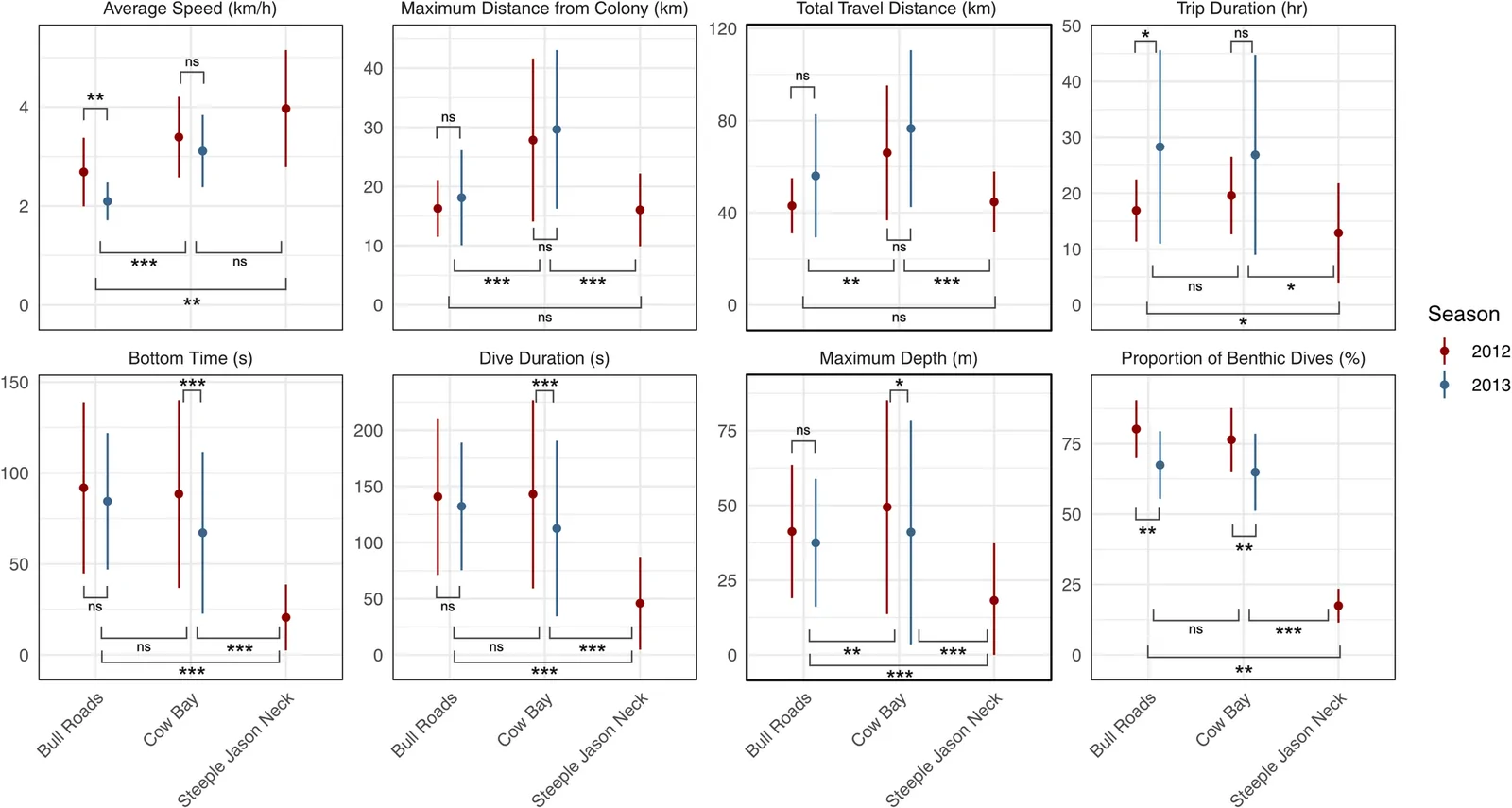
Mean and standard deviation of trip (upper row) and dive (bottom row) characteristics for gentoo penguins at the Falkland Islands from three colonies during the breeding period of 2012 (red) and 2013 (blue). Exact values, standard error, ranges, and model selection criteria are available in Supplementary Table 5. Distributions are based on the guard period of Bull Roads in 2012 (*n* = 14) and 2013 (*n* = 20), the guard period of Cow Bay in 2012 (*n* = 17) and 2013 (*n* = 19), and the incubation period of Steeple Jason Neck in 2012 (*n* = 8). Codes denote whether differences between colonies or seasons were significant at the *p* < 0.05 (*), *p* < 0.01 (**), or *p* < 0.001 (***) levels, or if no significant difference was found (ns). Graphs were produced with the *ggplot2* R package v4.0.3 (Wickham et al. 2016)

**Fig. 3:**
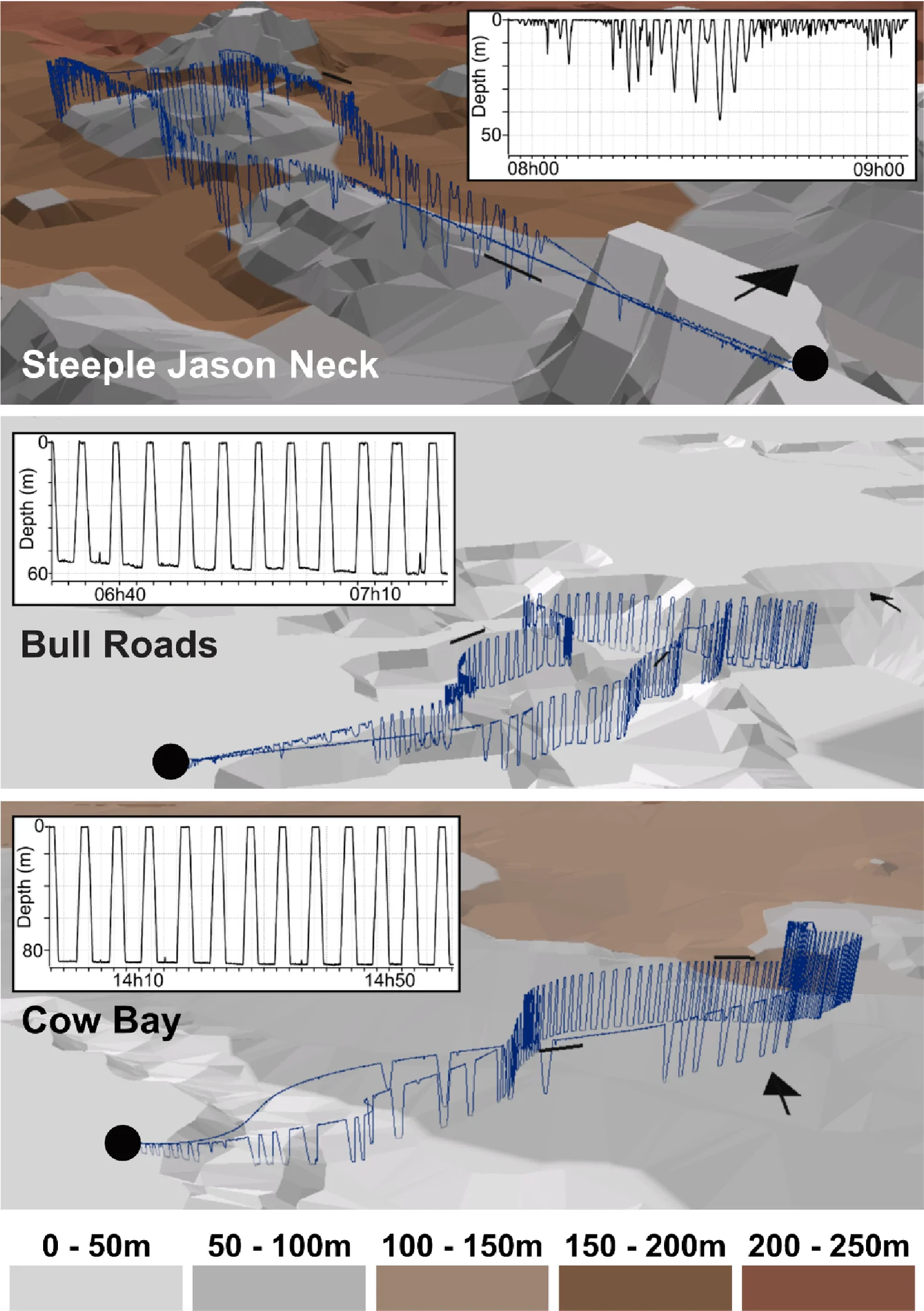
Three-dimensional representation (blue line) of a typical foraging trip from (top) Steeple Jason Neck, (middle) Bull Roads and (bottom) Cow Bay. Colony locations are represented by black circles, and insets show a portion of a cross-sectional typical dive profile. Black bars show 1 km scale at given location in image because scale in 3D varies in a linear perspective fashion. Arrows depict north. GPS locations were interpolated to the same time interval as TDR loggers (1s) to create tracks. Bathymetry data were sourced from the General Bathymetric Charts of the Oceans (GEBCO). Visualisation was achieved using ArcScene v10.3 (ESRI 2014)

Birds had different dive characteristics among the three colonies (Fig. 2; Supplementary Table 5). Birds from Bull Roads and Cow Bay during the guard period dived for longer and spent more time at the bottom of a dive than incubating birds from Steeple Jason. Similarly, penguins from Steeple Jason made the shallowest dives, with those from Bull Roads making dives of intermediate depth and those from Cow Bay diving to the deepest depths. Trips made by birds from Bull Roads and Cow Bay during the guard period featured a high proportion of benthic dives, and individuals reached sequentially deeper depths during the outbound phase of the journey followed by sequentially shallower dives during the inbound phase (Fig. 3).

Meanwhile, trips made by incubating penguins from Steeple Jason featured a much lower proportion of benthic dives (Fig. 2). The relative dive effort per cell (Fig. 1) indicates a consistent foraging effort, with a relatively uniform distribution of dive effort aside from in cells adjacent to the colony, which all birds must traverse early and late in their trips. Birds from Cow Bay spent more time at the bottom of a dive, reached deeper depths, dived for longer, and exhibited a greater proportion of benthic dives in 2012 than 2013 (Fig. 2). The only interannual difference in dive behaviour observed among birds from Bull Roads was a greater proportion of benthic dives in 2012 (Fig. 2).

Trip characteristics derived from GPS data also showed significant differences among colonies (Fig. 2; Supplementary Table 5). Travel speeds were fastest among incubating penguins from Steeple Jason, intermediate among those from Cow Bay during the guard period, and slowest among those from Bull Roads during the guard period. Birds from Steeple Jason also had shorter trip durations than those from Cow Bay and Bull Roads. Individuals from Cow Bay travelled further from the colony than those from Bull Roads and Steeple Jason. Bird from Bull Roads swam faster and undertook trips with shorter duration in 2012 than 2013 (Fig. 2). No meaningful difference in trip properties was observed between years among penguins from Cow Bay.

### Habitat use

Cross-validation performance metrics were strong for each presence-absence model (CBI > 0.76, AUC > 0.97, TSS > 0.88) and each diving intensity model (MAE < 0.07, RMSE < 0.11, R^2^ > 0.32) component of the hurdle models (Supplementary Tables 6 and 7).

**Fig. 4:**
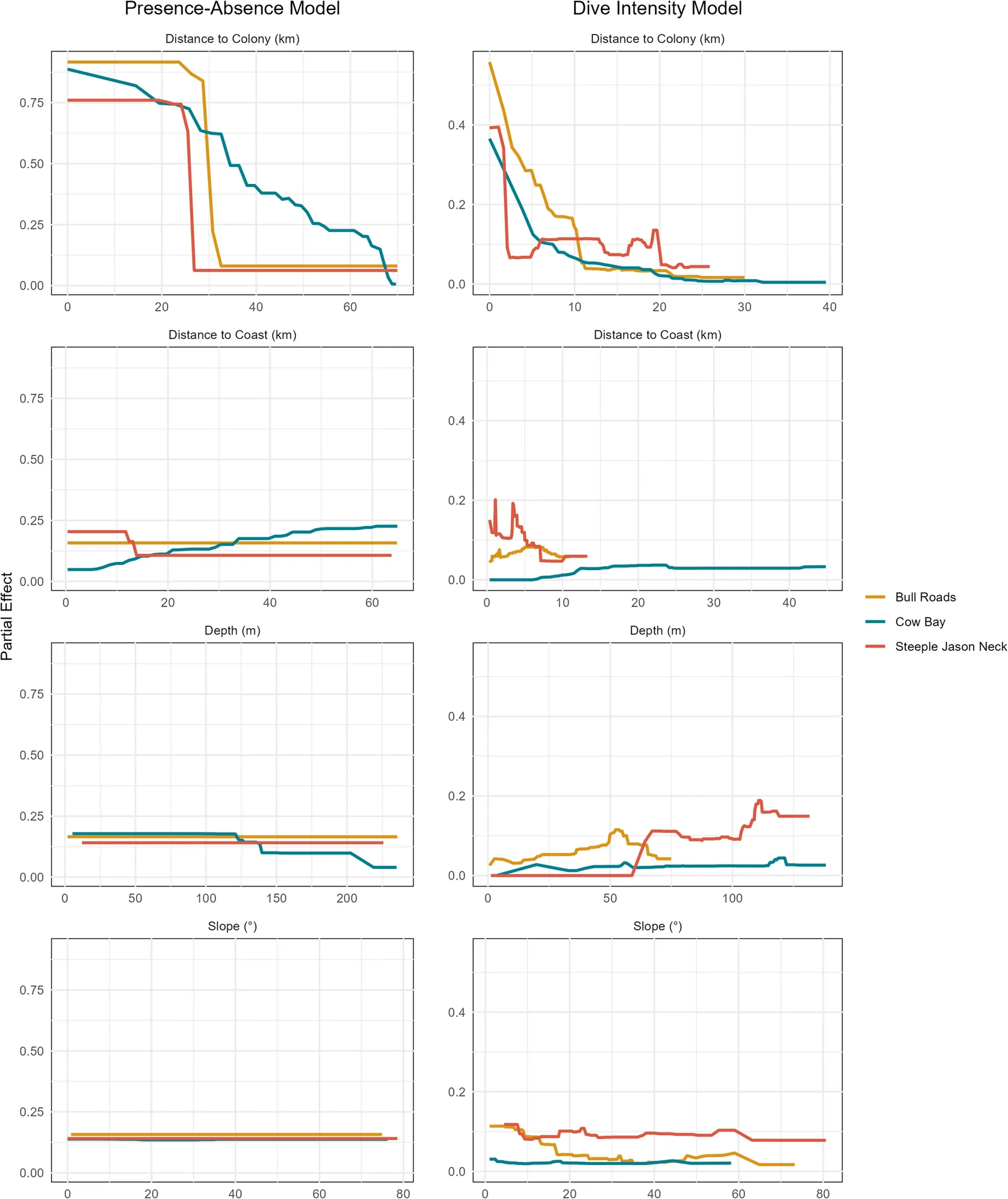
Partial dependence plots (PDPs) from the presence-absence model (left) and the dive intensity model (right) for the four environmental variables considered in models. Each colour corresponds to a different colony. Variables are ordered in approximate variable importance from top to bottom (see Supplementary Fig. 7). Graphs were produced with the ggplot2 R package v4.0.3 (Wickham et al. 2016)

Partial dependence plots (Fig. 4) depicted the varying habitat preferences of the three colonies. Distance to the colony was the most important variable in all models and for all colonies (Supplementary Fig. 7). In the presence-absence model, the partial effect declined for all colonies with increasing distance to the colony, though this was gradual decline for birds from Cow Bay but a sharp decrease between 25 km and 30 km around the other colonies (Fig. 4). Incubating birds from Steeple Jason exhibited a slight declining preference for areas further from the coast, but those from Cow Bay during the guard period demonstrated the opposite trend (Fig. 4). Depth had no impact on penguins from Bull Roads or Steeple Jason, but those from Cow Bay exhibited a slight preference for depths shallower than 125 m. Slope had negligible impact for any colony (Fig. 4).

The diving intensity model also indicated a decline in activity in regions further from the colony, though with greater variability than in the presence-absence model (Fig. 4). A general decline in suitability was observed with distance to coast for birds from Steeple Jason, while those from Bull Roads displayed a slight preference for distances of ~ 5 km, and those from Cow Bay demonstrated slightly increased diving intensity with increased distance from the coast (Fig. 4). Penguins from Bull Roads peaked in diving intensity at depths around 50 m, while those from Cow Bay exhibited little variability with depth, and those from Steeple Jason demonstrated a far greater diving intensity at depths above 70 m (Fig. 4). Individuals from Bull Roads showed the greatest diving intensity over shallower slopes, while those from Cow Bay and Steeple Jason exhibited little variability with slope (Fig. 4).

**Fig. 5:**
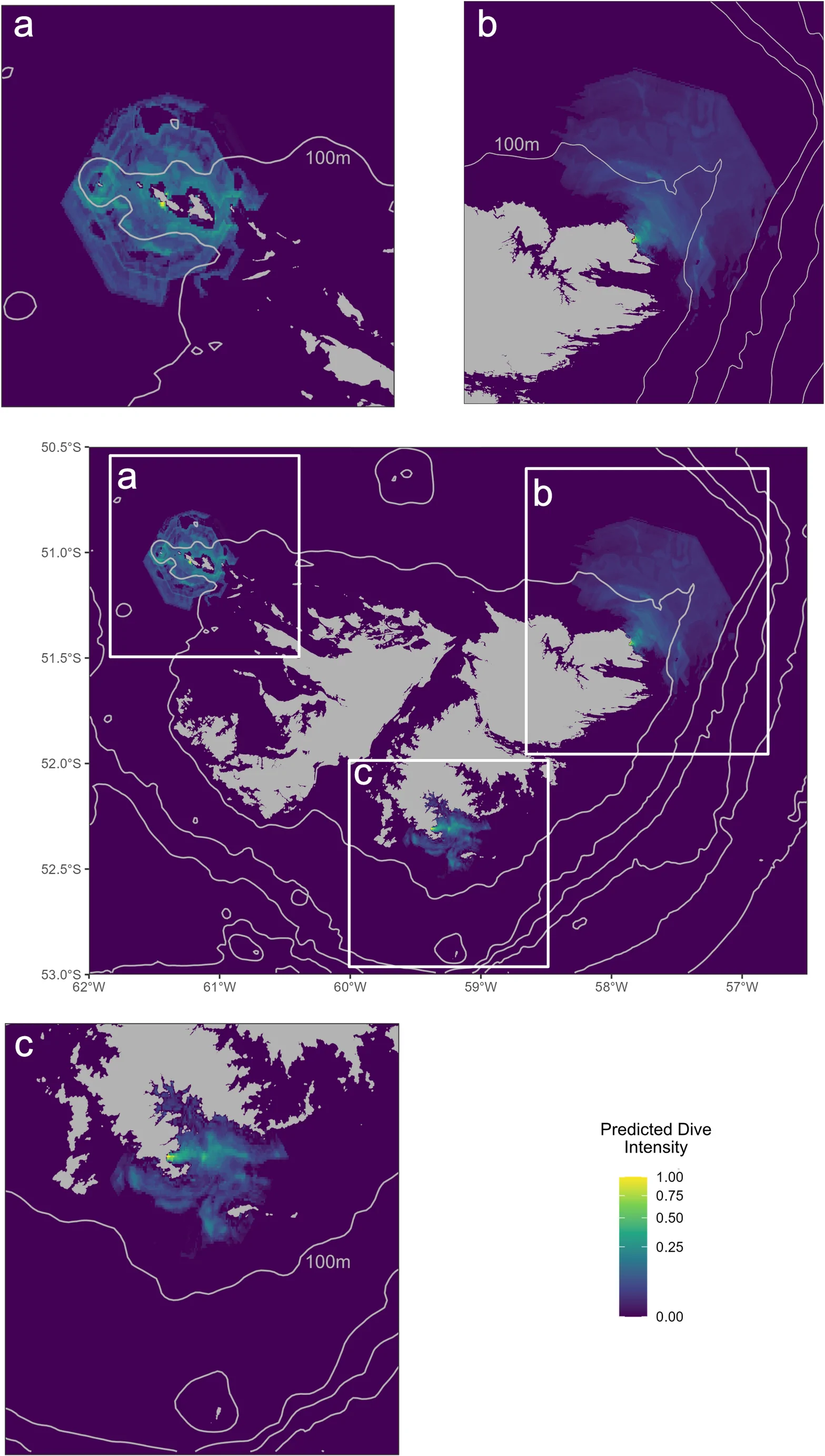
Predicted diving intensity for gentoo penguins at the Falkland Islands from the hurdle model. The central panel shows the output for the entire Falkland Islands, while the outer panels offer a closer view of suitability in the waters surrounding (**a**) Steeple Jason, (**b**) Cow Bay, and (**c**) Bull Roads. Isobaths denote depth contours at 100 m intervals, and the 100 m contour is labelled on outer panels. A square root transformation was applied to the colour scale for dive intensity to truncate values of cells adjacent to colonies, which were intensely used by penguins. Graphs were produced with the *terra* R package v1.9-1 (Hijmans et al. 2026). The Falkland Islands shapefile was obtained from the South Atlantic Environmental Research Institute (https://www.south-atlantic-research.org/webgis-page/)

The combined hurdle model (Fig. 5) predicted similar patterns of space use to the observed diving intensity (Fig. 1). Around Bull Roads, predicted dive intensity was greatest within the bay east of the colony, though other waters to the south and southeast of the colony were predicted to see greater dive intensity than observed (Fig. 5). Around Cow Bay, predicted dive intensity was moderate across the continental shelf, and higher in waters immediately adjacent to the colony (Fig. 5). Predicted dive intensity around Steeple Jason was high around the 100 m depth contour to the west of the island as observed but was also high in waters surrounding the Jason Islands, where comparatively lower diving intensity was observed (Fig. 5).

## Discussion

Our multi-year study, the largest tracking effort for gentoo penguins at the Falklands Islands, showed that gentoo penguins across the archipelago exhibited colony specific foraging behaviours, yet all birds foraged in waters near to their colonies. Site-specific variation in behaviour despite broadly consistent inshore foraging strategies reflect the combined influence of central-place foraging constraints with local habitat and prey availability. Penguins from Cow Bay and Bull Roads encountered gently sloping seabeds and primarily performed benthic dives, whereas those from Steeple Jason encountered steeply sloping seabeds and primarily performed pelagic dives. These results support the concurrent dietary investigation that revealed colony-specific differences in diet (Handley et al. 2017). Individuals from Cow Bay primarily fed on the rock cod (*Patagontothen spp.*), while those from Bull Roads ate lobster krill (*Munida spp.*), both of which are benthic organisms. Life history data for these prey items, given their size at consumption, suggest that their expected distribution is on or near the sea floor (Brickle et al. 2006; Meerhoff et al. 2013). Another concurrent investigation using animal-borne camera loggers at these two colonies confirmed that these birds foraged along the seafloor (Handley et al. 2018). At Steeple Jason, the main prey item was Falkland herring (*Sprattus fuegensis*), which is typically found in coastal, pelagic waters, where it schools at depths up to 70 m (Whitehead 1985).

Dive characteristics of gentoo penguins have previously been shown to differ between benthic and pelagic prey, and to vary with local bathymetry (Lescroël and Bost 2005; Kokubun et al. 2010; Miller et al. 2010; Carpenter-Kling et al. 2017; Lee et al. 2021). The strong influence of local habitat and prey availability on the foraging behaviour of gentoo penguins from the three different breeding colonies is likely enhanced by the limited range of these birds during the breeding period. Diving behaviour of other penguin species with a limited foraging range, such as the little penguin (*Eudyptula minor*) and yellow eyed penguin (*Megadyptes antipodes*), is similarly dictated by local bathymetry (Chiaradia et al. 2007; Mattern et al. 2007; Muller et al. 2020).

Some interannual variability was also observed among birds from Bull Roads and Cow Bay. The longer trips made by penguins from Bull Roads in 2013 coincide with a year of anomalously low breeding success, which was theorised to be linked with the unusually cool sea surface temperatures in that year (Stanworth 2013). The diet of these birds was also far less diverse in 2013 than 2012, feeding almost entirely on lobster krill, and consumed lobster krill were considerably smaller in 2013 than 2012 (Handley et al. 2017). Perhaps the cooler waters of 2013, indicative of broader oceanographic conditions, coincided with a period of reduced prey availability, necessitating longer foraging trips. Among birds from Cow Bay, trip characteristics did not change, but a greater proportion of dives were pelagic in 2013 than 2012, corresponding with shallower, shorter dives. Breeding success was stable between these years (Stanworth 2013), but the diet of these birds in 2013 included a greater proportion of the pelagic southern blue whiting (*Micromesistius australis*) (Handley et al. 2017), which could explain why there were a greater proportion of pelagic dives that year. Again, it is possible that the cooler temperatures in 2013 were linked with prey availability, in this case providing a greater availability of pelagic prey. These interannual changes further highlight the flexibility of gentoo penguin foraging behaviour.

Gentoo penguins from Cow Bay foraged over an area four times larger than that used by the other two colonies. A smaller foraging range was observed among penguins from Steeple Jason, though these tracks were obtained during incubation, and the breeding stage could possibly influence foraging behaviour. That said, incubation trips are generally longer than those during brood guard for seabirds, including gentoo penguins (Otley et al. 2004; Oppel et al. 2018), due to the provisioning of young chicks requiring frequent returns to the colony. Therefore, it is unlikely that trips made during brood guard from Steeple Jason would be considerably longer. However, it should be noted that gentoo penguins from a colony in South Georgia used smaller foraging ranges during incubation than during guard (Ratcliffe et al. 2018), showing that this is still a possibility. While breeding stage may influence trip characteristics, diving behaviour is unlikely to vary much between incubation and guard at Steeple Jason due to the scarcity of shallow slopes for benthic foraging. It is also possible that the small sample size of eight individuals from Steeple Jason does not fully represent the range of behaviours exhibited by the entire population. However, given the comparatively small standard deviation of trip and dive metrics at Steeple Jason (Fig. 2), this population may be more homogenous, particularly since smaller sample sizes are generally associated with greater uncertainty. The smaller foraging range at Bull Roads could be an artefact of reduced intra-specific competition owing to smaller colony sizes (Patterson et al. 2022), or because of the physical constraints placed on the colony through its position at the head of a bay (Chiaradia et al. 2007).

Competition between colonies could also limit the foraging ranges at Steeple Jason and Bull Roads, where some of the nearby predicted suitable diving habitat was relatively unused. Much of this unused suitable habitat, such as that to the south of Bull Roads, was located close to other gentoo penguin colonies. It could be that spatial resource partitioning between adjacent colonies limits the area available around these colonies, as observed elsewhere (Masello et al. 2010; Lee et al. 2021). Other penguin species, such as Magellanic penguins (*Spheniscus magellanicus*) and southern rockhopper penguins (*Eudyptes chrysocome*), also reside at nearby colonies and might influence foraging behaviour. However, these species typically forage further offshore than gentoo penguins (Baylis et al. 2019), which would reduce spatial overlap. It should however be noted that there was some evidence of overfitting in the BRTs, as demonstrated by fluctuating relationships in the PDPs, which could bias these predictions.

The diverse foraging behaviours observed at different gentoo penguin colonies could mean that some colonies fare better than others when exposed to differing environmental conditions. Alternatively, differences in foraging behaviours could enable gentoo penguins to successfully adapt to environmental variability. In the southernmost limits of their range, on the Antarctic Peninsula and South Shetland Islands, gentoo penguins have maintained high levels of breeding and foraging success through highly variable environmental conditions (Juáres et al. 2020; Herman et al. 2020; Machado-Gaye et al. 2025). However, in other northerly locations, such as Macquarie Island and the Prince Edward Islands, populations have declined despite observed variation in foraging behaviour (Crawford et al. 2014; Carpenter-Kling et al. 2019; Pascoe et al. 2020). Broadly speaking, generalist species are faring better than specialists in the face of global change (MacLean and Beissinger 2017). Despite this, generalist species can still experience environmental stress. Indeed, gentoo penguin population dynamics in the Falkland Islands are tied to environmental variables, as there are fewer breeding pairs in years with lower Southern Oscillation Index values and associated warmer spring sea surface temperatures (Baylis et al. 2012).

In light of ongoing marine spatial planning efforts across the Falkland Islands, a developing hydrocarbon industry, and existing commercial fisheries (Augé et al. 2018; Baylis et al. 2021; Handley et al. 2023), our results provide critical evidence for informed decision making. Specifically, by linking movement, foraging behaviour, and local environmental conditions, this study highlights that gentoo penguins exhibit flexible foraging behaviours but remain largely restricted to nearshore areas. This limited foraging range makes gentoo penguins potentially vulnerable to nearshore threats, including commercial fisheries, pollution, and habitat disturbance. One way to mitigate these pressures is through site-specific measures such as marine protected areas that can help safeguard key foraging habitats and enhance colony resilience to both anthropogenic and natural threats. Future studies could complement this work by investigating how foraging behaviours within colonies vary temporally in relation to climatic indices, such as the Southern Oscillation Index, and demographic changes, such as the recent mass mortality event caused by the highly pathogenic avian influenza outbreak (Kuepfer and Stanworth 2024).

## Supplementary Information

Below is the link to the electronic supplementary material.

Supplementary Material 1

## Data Availability

All code used for statistical analyses and figure creation are available at https://github.com/oshuwilson/falklands-gentoos. GPS tracking data are available from the BirdLife International Seabird Tracking Database at https://data.seabirdtracking.org/dataset/2558, https://data.seabirdtracking.org/dataset/2559, and https://data.seabirdtracking.org/dataset/2560.
