# Supplementary material for: Multi-colony tracking of a marine central place forager reveals a site specific yet broadly consistent foraging strategy
Source: Mar Biol. 2026 Jul 15;173(9):149. doi: 10.1007/s00227-026-04909-3 (PMC13372836; doi:10.1007/s00227-026-04909-3)
Supplement: Supplementary file 1 — Supplementary Material 1 [file 227_2026_4909_MOESM1_ESM.pdf]

Supplementary Information

Linear Mixed Effects Models 2
Dynamic Covariates 2
Hurdle Model Components 5
Deployment Success 6
Sample Representativeness 7
At-Sea Distribution 9
Trip and Dive Characteristics 10
Hurdle Model Performance 11

Dynamic Environmental Conditions

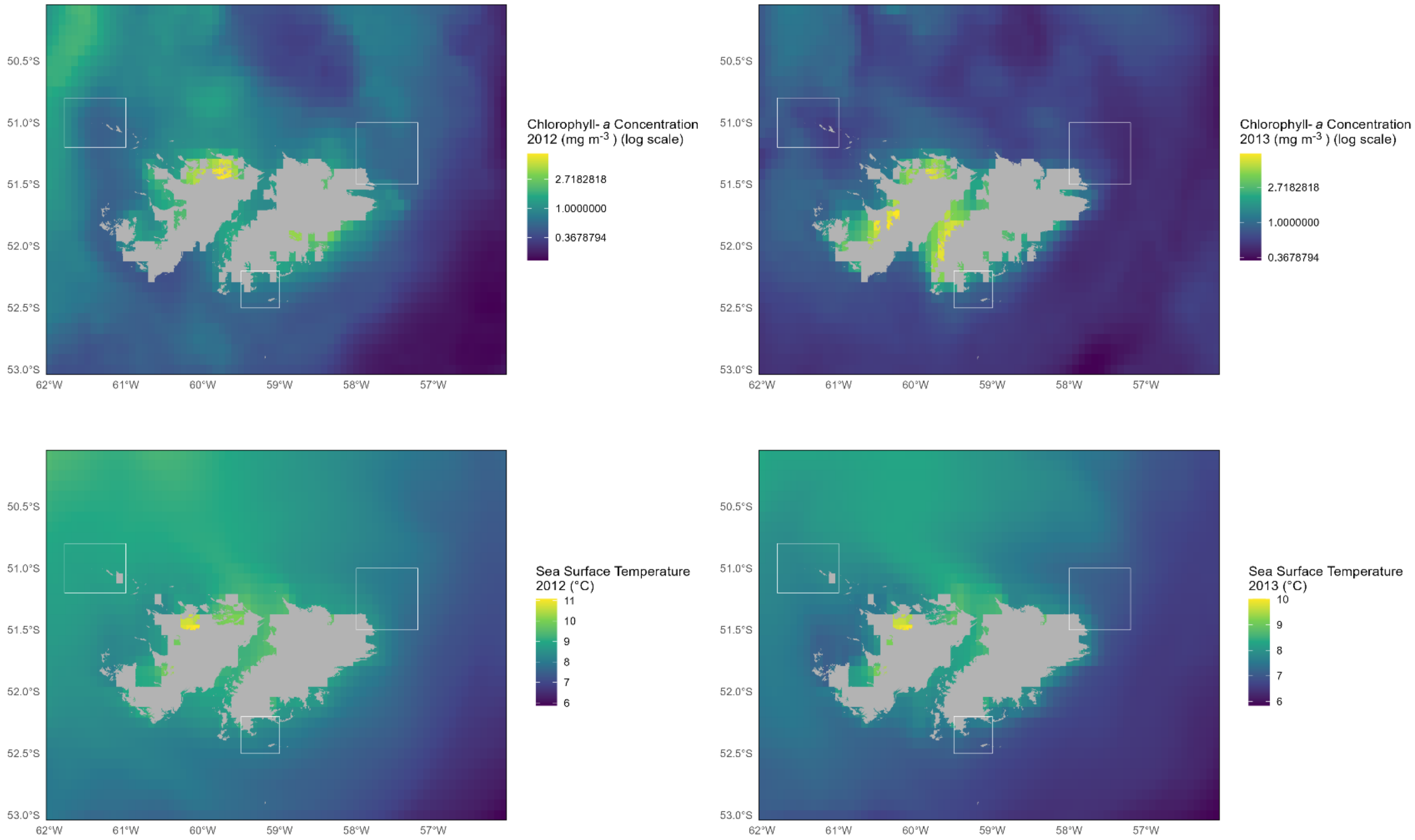

Supplementary Figure 1 | Example dynamic variables for the breeding seasons 2012/13 (left) and 2013/14 (right). White boxes are bounding boxes containing the maximum and minimum latitudes and longitudes of tracks from each of the three colonies. Colour scales for chlorophyll-*a* concentrations are log transformed. Rasters presented here are monthly rasters taken from December of the study year, though values derived in Supplementary Table 2 are derived from daily rasters averaged over each day of the respective tracking period for that colony. The resolution of these rasters is 0.083° (~ 9km latitude, ~ 6 km longitude in the study area).

Supplementary Table 1 | Mean and standard deviation of dynamic variables within the bounding boxes for each colony presented in Supplementary Figure 2, and across the whole study area, bounded by the maximum longitude and latitude of all track locations and inclusive of the colony bounding boxes. Italicised values were statistically distinct from the corresponding value in another year at the p < 0.001 level, as per Wilcoxon-rank sum tests.

	**Chlorophyll-*a* Concentration (mg m^{-3})**		**Sea Surface Temperature (°C)**	
**Colony**	**2012 Mean (SD)**	**2013 Mean (SD)**	**2012 Mean (SD)**	**2013 Mean (SD)**
Bull Roads	*0.71 (0.16)*	*1.02 (0.4)*	*8.17 (0.24)*	*7.18 (0.25)*
Cow Bay	0.62 (0.08)	0.6 (0.12)	*8.02 (0.23)*	*7.12 (0.23)*
Steeple Jason	0.67 (0.16)	-	8.84 (0.06)	-
**Whole Area**	*0.67 (0.49)*	*0.77 (0.63)*	*8.09 (0.79)*	*7.28 (0.66)*

Linear Mixed Effects Models

Model Selection

Supplementary Table 2 | Model selection justification for the inclusion of colony as a fixed effect in all linear mixed effects models used to compare diving and trip characteristics. The model including colony is compared with a null model which featured no fixed effects. Models with the lowest AIC, lowest BIC, and highest log likelihood (logLik) are highlighted in bold for their respective columns. L-Ratios and p-values are also reported from results of ANOVA models used to determine if models offered any meaningful improvement in evaluation metrics over null models.

Model Fixed Effect	AIC	BIC	logLik	L-Ratio	p-value
Dive Duration (s)					
Colony	**354859.7**	**354927.2**	**-177421.9**	70.695	< 0.0001
Null	354926.4	354977.0	-177457.2		
Maximum Depth (m)					
Colony	**293299**	**293366**	**-146641**	59.321	< 0.0001
Null	293354	293404	-146671		
Bottom Time (s)					
Colony	**305988.8**	**306055.9**	**-152986.4**	85.213	< 0.0001
Null	306070.1	306120.4	-153029.0		
**Proportion Benthic (%)**					
Colony	**908.13**	**927.34**	**-447.06**	71.009	< 0.0001
Null	975.14	988.86	-482.57		
Average Speed (kmh^{-1})					
Colony	**196.73**	**213.66**	**-91.37**	29.07	< 0.0001
Null	221.80	233.90	-105.90		
Trip Duration (hrs)					
Colony	**679.81**	**696.74**	**-332.9**	6.94	0.031
Null	682.74	**694.84**	-336.37		
Max Distance (km)					
Colony	**619.42**	**636.36**	**-302.71**	22.85	< 0.0001
Null	638.28	650.37	-314.14		
Path Length (km)					
Colony	**782.08**	**799.01**	**-384.04**	16.88	< 0.0001
Null	794.96	807.05	-392.48		

Dynamic Predictor Inclusion

Supplementary Table 3 | Model performance metrics for presence-absence and dive intensity models that only use static predictors and those that also use dynamic predictors. Presence-absence models were assessed with the Continuous Boyce Index (CBI), which is scored from -1 to 1. Values closer to 1 indicating better performance for the CBI. Dive intensity models were assessed using Mean Absolute Error (MAE), which can take any non-negative value, and values closer to 0 indicate better performance.

Colony	Static Presence-Absence CBI	Dynamic Presence-Absence CBI	Static Intensity MAE	Dynamic Intensity MAE
**Bull Roads**	0.934	0.712	0.032	0.036
**Cow Bay**	0.963	0.787	0.014	0.02
**Steeple Jason**	0.763	0.711	0.069	0.076

Hurdle Model Components

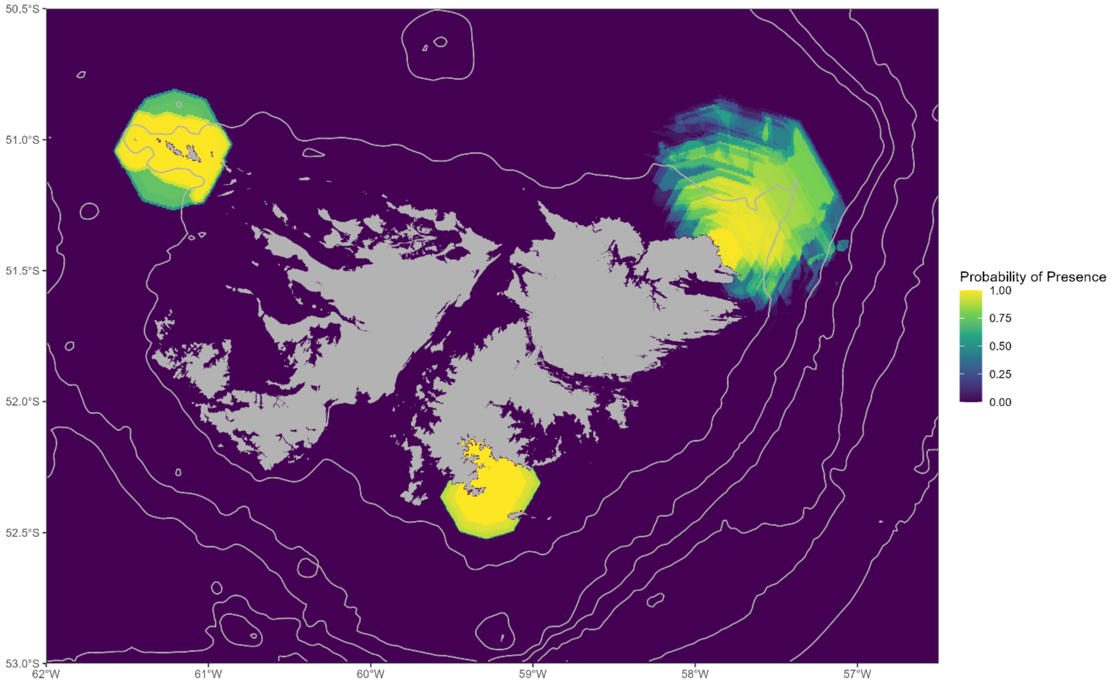

Supplementary Figure 2 | Predicted probability of presence from the first component of the hurdle model only.

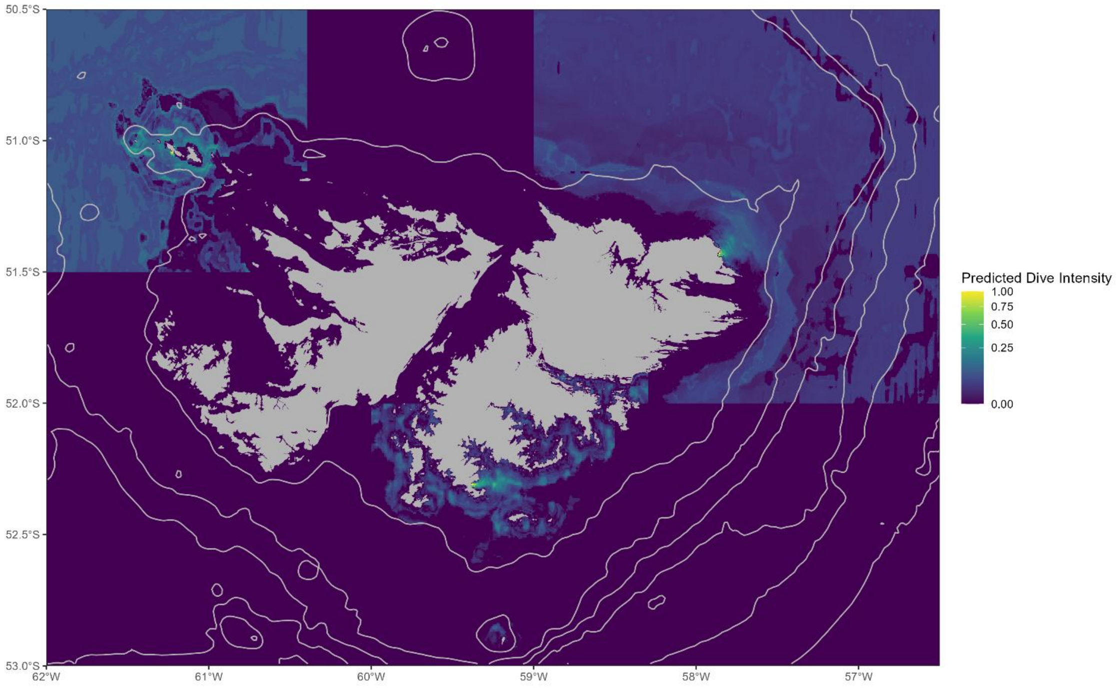

Supplementary Figure 3 | Predicted dive intensity from the second component of the hurdle model only.

Deployment Success

Supplementary Table 4 | Overview of the deployment success for the 103 birds equipped with both GPS and TDR units during the study. Of these, batteries from two deployments failed, four birds were not recaptured, and nine devices failed owing to water penetration. A test for sample representativeness indicated that too few tracks were obtained for the incubation period of 2012 at Cow Bay (n=5) and Bull roads (n=5). Sampling periods are defined by site-season-breeding stage. SJ, BR, CB: Steeple Jason Neck, Bull Roads, Cow Bay. 1213, 1314: 2012/13, 2013/14. I, G: Incubation, Guard.

Sampling periods	Deployments for analysis	Sample too small	Battery failure	Water penetration	Birds uncaptured	Total deployments
SJ-1213-I	8			4	2	14
BR-1213-I		5				5
BR-1213-G	14			2	2	18
BR-1314-G	20					20
CB-1213-I		5		3		8
CB-1213-G	17		2			19
CB-1314-G	19					19

Sample Representativeness

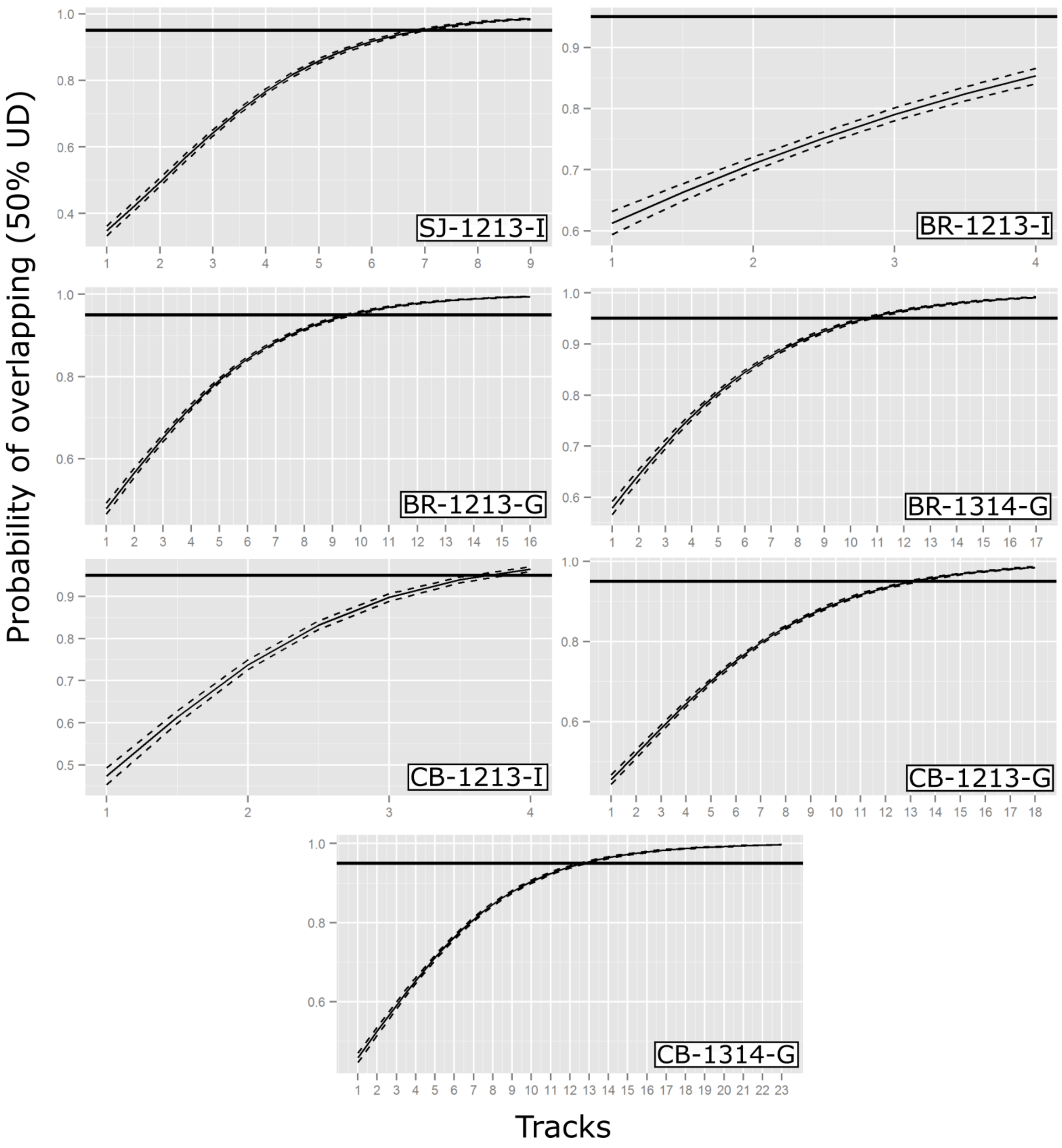

Supplementary Figure 4 | Probability of tracks overlapping at the 50% utilization distribution. Dashed lines indicate upper and lower confidence intervals associated with the curve after 500 bootstraps. Iterations were performed from 1 to n-1 tracks, where n is the number of tracks from a given sampling period. Solid horizontal line indicates the 95% probability of overlapping. Sampling periods are defined by site-season-breeding stage. SJ, BR, CB: Steeple Jason Neck, Bull Roads, Cow Bay. 1213, 1314: 2012/13, 2013/14. I, G: Incubation, Guard.

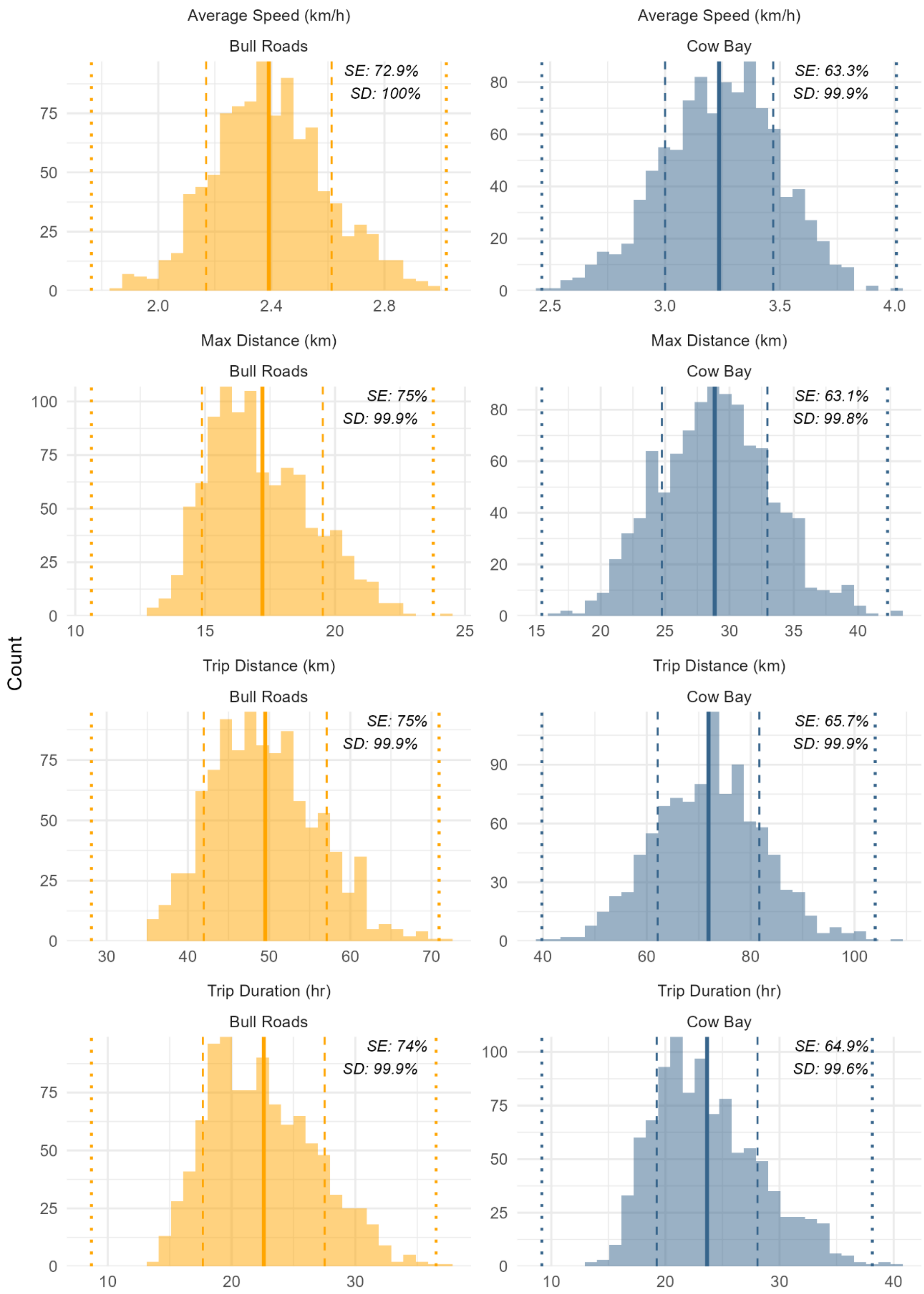

Supplementary Figure 5 | Distribution of mean values for trip characteristics among birds from Bull Roads (orange) and Cow Bay (blue) when using 1000 bootstrapped samples of eight individuals, equivalent to the number of tagged individuals from Steeple Jason. The solid vertical line represents the complete sample mean, the dashed lines denote the complete sample standard error, and the dotted lines denote the complete sample standard deviation. Italicised text reports the proportion of bootstrapped samples that fall within the standard error (SE) and standard deviation (SD) of the full sample.

At-Sea Distribution

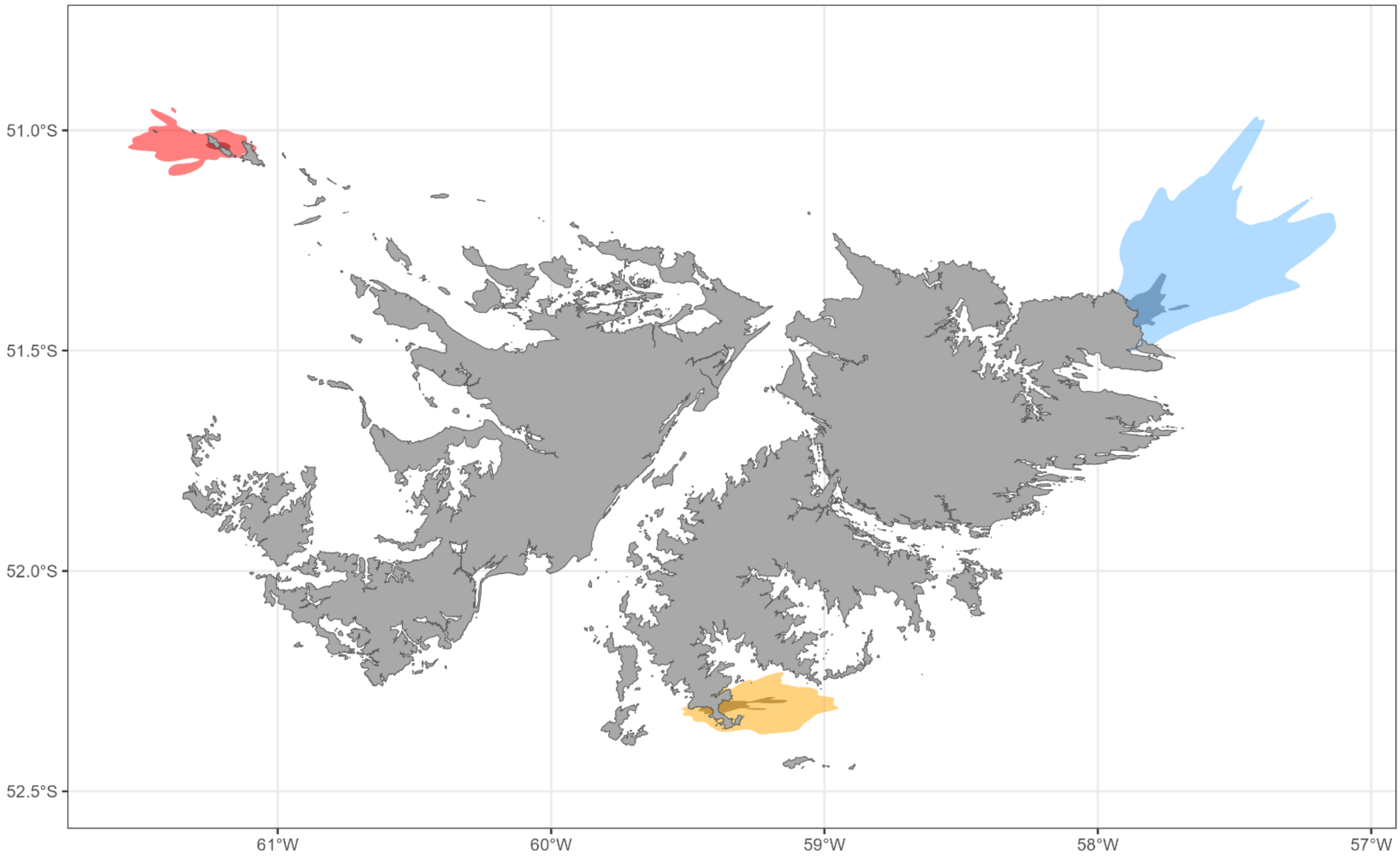

Supplementary Figure 6 | 50% (dark) and 95% (light) utilization distributions for breeding gentoo penguins from Bull Roads (orange), Cow Bay (blue), and Steeple Jason (red).

Trip and Dive Characteristics

Supplementary Table 5 | Mean (SE) and range of dive and trip characteristics for Gentoo Penguins at the Falkland Islands from three colonies (BR: Bull Roads, CB: Cow Bay, SJN: Steeple Jason Neck) during the breeding period (Incubation, Guard) of 2012 and 2013.

	**BR 2012 (Guard)**		**BR 2013 (Guard)**		**CB 2012 (Guard)**		**CB 2013 (Guard)**		**SJ 2012 (Incubation)**	
	**Mean (SE)**	**Range**	**Mean (SE)**	**Range**	**Mean (SE)**	**Range**	**Mean (SE)**	**Range**	**Mean (SE)**	**Range**
**Dive Characteristics**										
Dive duration (s)	140.8 (4.63)	1 - 294	132.2 (4.31)	1 - 257	143 (4.86)	1 - 307	112.4 (3.19)	1 - 318	48.2 (7.65)	1 - 193
Maximum depth (m)	41.2 (1.39)	3 - 75	37.5 (1.35)	3 - 77.4	49.4 (1.52)	3 - 152.2	41.1 (1.43)	3 - 140.2	19 (2.47)	3 - 102.2
Bottom time (s)	87.9 (4.25)	1 - 214	82.3 (4.12)	1 - 194	87.6 (3.81)	1 - 225	69.6 (3.34)	1 - 221	20.2 (5.04)	1 - 130
**Trip characteristics**										
Proportion benthic (%)	80.4 (2.62)	52.9 - 92	66.8 (2.63)	43.9 - 89.6	76.5 (2.65)	50.2 - 94.8	64.6 (2.48)	43.1 - 92.6	18.1 (1.76)	10.6 - 35.5
Avg. travel speed (kmh^{-1})	2.69 (0.14)	1.37 - 4	2.1 (0.14)	1.52 - 2.98	3.39 (0.18)	1.33 - 4.83	3.1 (0.17)	1.32 - 4.58	3.97 (0.42)	1.68- 5.33
Trip duration (hrs)	16.91 (3.22)	6.1 - 28.7	28.29 (3.22)	10.1 - 77.8	19.59 (3.25)	5.6 - 31.8	26.86 (2.89)	8.7 - 85.9	12.9 (3.14)	6.5 - 34.5
Max distance (km)	16.3 (1.66)	8.1 - 28.1	18.1 (1.66)	9.1 - 44.4	27.8 (3.11)	12.2 - 58.4	30.3 (2.95)	8.5 - 58.7	16.1 (2.18)	6.9 - 23.3
Path length (km)	43 (5.17)	23.1 - 67.2	56 (5.17)	26.4 - 118.4	66 (7.35)	19.8 - 121.9	76.5 (6.54)	19.9 - 143.2	44.7 (4.66)	22 - 58

Hurdle Model Performance

Supplementary Table 6 | Hyperparameter options selected following tuning for use in final presence-absence models along with associated model performance metrics. Performance metrics include the Continuous Boyce Index (CBI), Area Under the Receiver Operating Curve (AUC) and True Skill Statistic (TSS). The CBI is scored from -1 to 1 and AUC and TSS are scored from 0 to 1. Values closer to 1 indicating better performance for all three metrics.

Colony	Trees	Tree Depth	Learning Rate	CBI	AUC	TSS
**Bull Roads**	500	1	0.005	0.934	0.986	0.883
**Cow Bay**	500	3	0.01	0.963	0.984	0.901
**Steeple Jason**	500	1	0.005	0.763	0.979	0.931

Supplementary Table 7 | Hyperparameter options selected following tuning for use in final dive intensity models along with associated model performance metrics. Performance metrics include the Mean Absolute Error (MAE), Root Mean Squared Error (RMSE) and Coefficient of Determination (R^2). MAE and RMSE can take any non-negative value, with values closer to 0 indicating better performance. R^2 is scored from 0 to 1, with values closer to 1 indicating better performance.

Colony	Trees	Tree Depth	Learning Rate	MAE	RMSE	R^2
**Bull Roads**	2000	3	0.01	0.032	0.057	0.576
**Cow Bay**	2000	3	0.01	0.014	0.029	0.406
**Steeple Jason**	2000	3	0.01	0.069	0.106	0.324

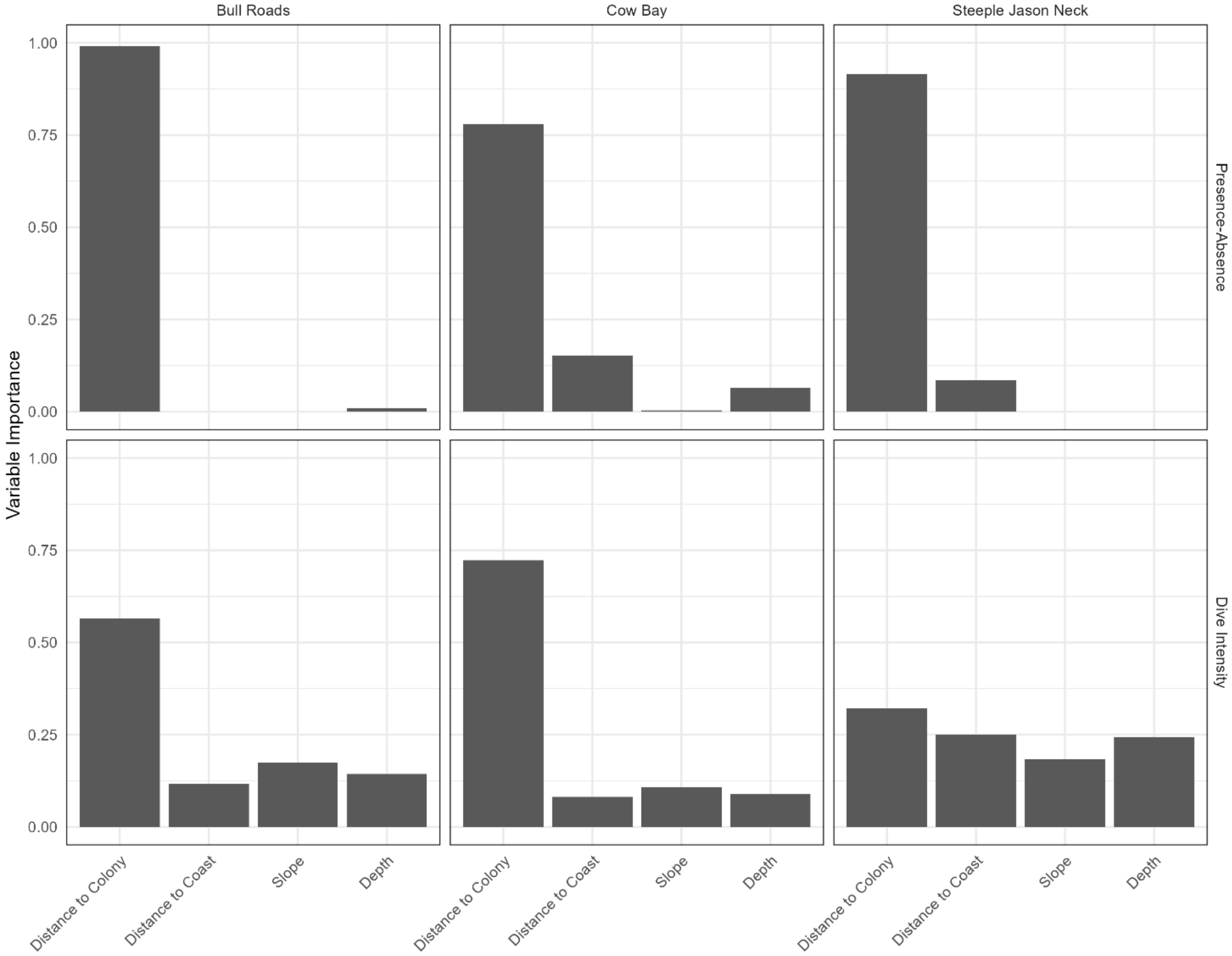

Supplementary Figure 7 | Variable importance scores from boosted regression trees for each colony in the presence-absence model (top) and the dive intensity model (bottom). Variables are ordered by average contribution. Empty bars are where variables did not exert any effect on predictions.
